# TGFβ induced factor homeobox 1 promotes colorectal cancer development through activating Wnt/β-catenin signaling

**DOI:** 10.18632/oncotarget.19603

**Published:** 2017-07-26

**Authors:** Ji-Lian Wang, Zhen Qi, Ye-Hua Li, Hong-Mei Zhao, Ye-Guang Chen, Wei Fu

**Affiliations:** ^1^ Department of General Surgery, Peking University Third Hospital, Beijing 100191, China; ^2^ The State Key Laboratory of Membrane Biology, Tsinghua-Peking Center for Life Sciences, School of Life Sciences, Tsinghua University, Beijing 100084, China

**Keywords:** colorectal cancer, TGIF1, Wnt signaling, β-catenin, TGF-β

## Abstract

Colorectal cancer (CRC) is one of the most common cancers, but the mechanisms underlying its initiation and progression are largely unknown. TGIF1 (TGFB induced factor homeobox 1) is a transcriptional corepressor that belongs to the three-amino acid loop extension (TALE) superclass of atypical homeodomains. It has been reported that TGIF1 is highly expressed in mammary cancer and non-small cell lung cancer and can enhance tumor progression. However, the role of *TGIF1* in colorectal cancer remains unknown. Here, we report that TGIF1 is significantly upregulated in colorectal cancers, and its high expression predicts poor prognosis. Overexpression of TGIF1 markedly promotes the proliferation of colorectal cancer cells both *in vivo* and *in vitro*. In addition, TGIF1 activates Wnt/β-catenin signaling, and the homeodomain is indispensable for Wnt activation and β-catenin interaction. Taken together, our results suggest that TGIF1 is a novel colorectal tumor promoter and indicate that TGIF1 enhances colorectal cancer tumorigenesis through activating Wnt signaling.

## INTRODUCTION

Colorectal cancer (CRC) is one of the most common cancers and has a high mortality rate in its later stage [1]. However, the molecular mechanisms underlying colorectal carcinogenesis are still not fully understood. Several factors are associated with the initiation and progression of CRC, including mutations in proto-oncogenes or tumor suppressor genes [2, 3], chromosomal instability and microsatellite instability [4–6] and epigenetic modifications [7]. The canonical Wnt signaling pathway is aberrantly activated in the majority of CRC patients due to mutations in adenomatous polyposis coli (APC*)* or β-catenin, and it is shown that hyperactivation of Wnt signaling plays an essential role in the pathogenesis of CRC [8, 9]. β-Catenin is the key mediator of the canonical Wnt signaling. In the absence of Wnt ligands, the destruction complex containing APC, Axin2, glycogen synthase kinase 3β (GSK-3β), casein kinase 1, and beta-transducin repeats-containing protein (β-TrCP) in the cytoplasm, phosphorylates and ubiquitinates β-catenin, leading to its proteasomal degradation [10, 11]. The binding of Wnt ligands to the receptor Frizzled and the coreceptor low density lipoprotein receptor-related protein 5 or 6 (LRP5/6) leads to dissociation of the destruction complex and thus β-catenin accumulation. Then, β-catenin translocates into the nucleus and acts with members of the T cell factor/lymphoid enhancer binding factor (TCF/LEF) family to activate transcription of downstream genes including *CCND1* and *c-Myc,* both of which are the cell cycle regulators upregulated in colorectal tumors [12–15]. Activation of Wnt signaling by gene mutations plays a crucial role in colorectal cancer development. Furthermore, some of the Wnt target genes also contribute to cancer progression [16].

TGIF1 (TGFB induced factor homeobox 1) is a member of the three-amino acid loop extension (TALE) superclass of atypical homeodomains and functions as a transcriptional corepressor to inhibit TGF-β signaling [17]. Mutations of the *TGIF1* gene are associated with holoprosencephaly, a severe human genetic disease affecting craniofacial development [18, 19]. The most known function of TGIF1 is the repression of TGF-β signaling by recruiting mSin3A and histone deacetylases (HDACs) to the TGF-β-activated Smad complex or targeting Smad2 for degradation or sequestration [20–24]. A number of studies have indicated the TGF-β/Smad-independent function of TGIF1, which are mediated by its direct DNA binding ability. For instance, TGIF1 inhibits the retinoic acid (RA) signaling pathway by binding to the retinoid X receptor response element and repressing transcription through DNA-binding competition [20–22, 25], and it negatively regulated MLL-rearranged acute myeloid leukemia by interfering with MEIS1 transcription through competing for DNA binding to a common motif [26]. Consistent with its important roles in regulating various signaling pathways, TGIF1 has important functions in embryonic development, stem cell quiescence and self-renewal and tumorigenesis [27–29]. TGIF1 has been showed to promote development of mammary cancer and non-small cell lung cancer and molecular mechanisms are still not fully understood [30, 31]. However, its role in CRC remains unknown.

This study aimed to reveal the expression pattern of TGIF1 in colorectal cancer and examine the function of TGIF1 in the progression of CRC. Our data indicate that TGIF1 is significantly overexpressed in CRC tissues and refers to poor prognosis. Moreover, TGIF1 plays an important role in promoting cancer cell proliferation and migration. We further show that TGIF1 functions through activating Wnt/β-catenin signaling, which is mediated by its DNA binding ability and interaction with *β-catenin*.

## RESULTS

### TGIF1 is highly expressed in CRC

**Figure 1:**
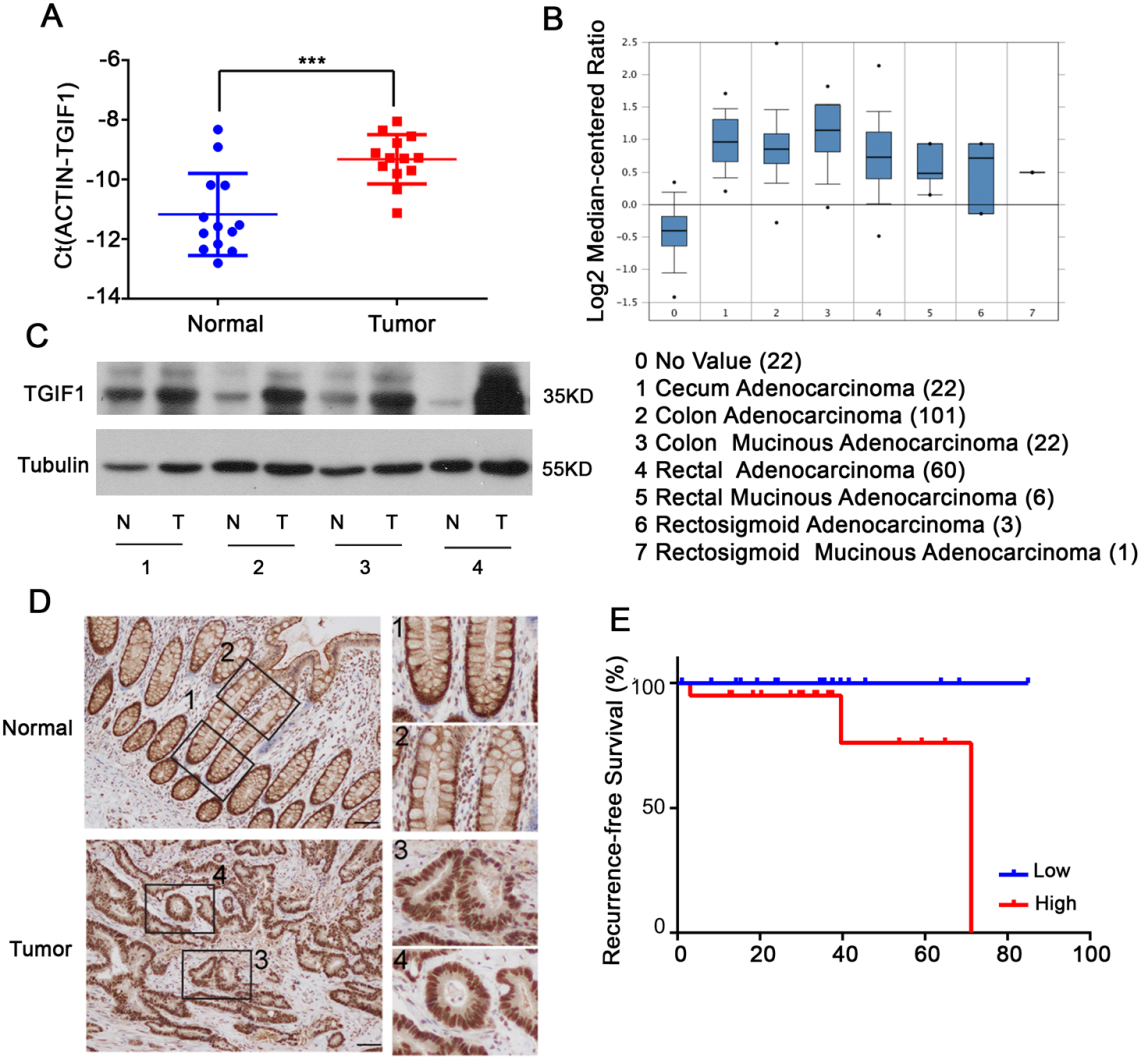
TGIF1 is highly expressed in CRC **(A)** qRT-PCR analysis of TGIF1 mRNA in CRC tissues (Tumor) and paired normal tissues (Normal). **(B)** TGIF1 expression analysis in all kinds of CRC according to Oncomine Database (https://www.oncomine.org/resource/login.html). **(C-D)** TGIF1 protein level in CRC tissues (T) and paired normal tissues (N) were assessed by immunoblotting (C) and immunohistochemistry (D) respectively. Enlargements in right: 1, stem cell region of normal crypts; 2, differentiation region of normal crypts; 3, 4, colon tumor regions. **(E)** Kaplan-Meier graph representing the probability of cumulative recurrence-free survival in CRC patients according to the TGIF expression in TCGA database, low (20) versus high (20). *p=*0.086.

We firstly compared *TGIF1* mRNA levels in CRC tissues and paired normal tissues by quantitative real-time PCR (qRT-PCR). Notably, CRC tissues showed significantly higher *TGIF1* expression than the paired normal tissues (Figure 1A). To confirm these findings, we firstly examined the expression pattern of *TGIF1* in the Oncomine database, and found that the overall mRNA levels of TGIF1 in CRC tissues were significantly higher than these in the paired normal tissues (Figure 1B). We further randomly chose four paired tissue samples to assess TGIF1 protein levels by immunoblotting and observed the increased TGIF1 protein levels in CRC tissues (Figure 1C). These results were confirmed by immunohistochemistry. TGIF1 exhibited a strong nuclear signal in almost every cancer cells (Figure 1D). However, the strong nuclear localization of TGIF1 can only be detected in normal stem/progenitor cells at the base of crypt but not in differentiated cells at the upper part of crypt (Figure 1D, Box1 and 2 vs. Box 3 and 4). These results indicate that the nuclear TGIF1 may promote stemness of stem/progenitor cells at the base of crypts and enhance their tumorigenic ability during transformation. Finally, we investigated whether the TGIF expression is correlated with the outcome of CRC patients. To do this, we made use of data from The Cancer Genome Atlas (TCGA, https://cancergenome.nih.gov/), which enabled us to generate two groups with “high” and “low” TGIF1 expression. Elevated expression of TGIF1 was correlated with poor patient survival (Figure 1E). Taken together, our results indicate the TGIF1 is significantly upregulated in CRC tissues and may play important roles in tumorigenesis.

### TGIF1 enhances proliferation, migration and clonogenicity of colon cancer cells

**Figure 2:**
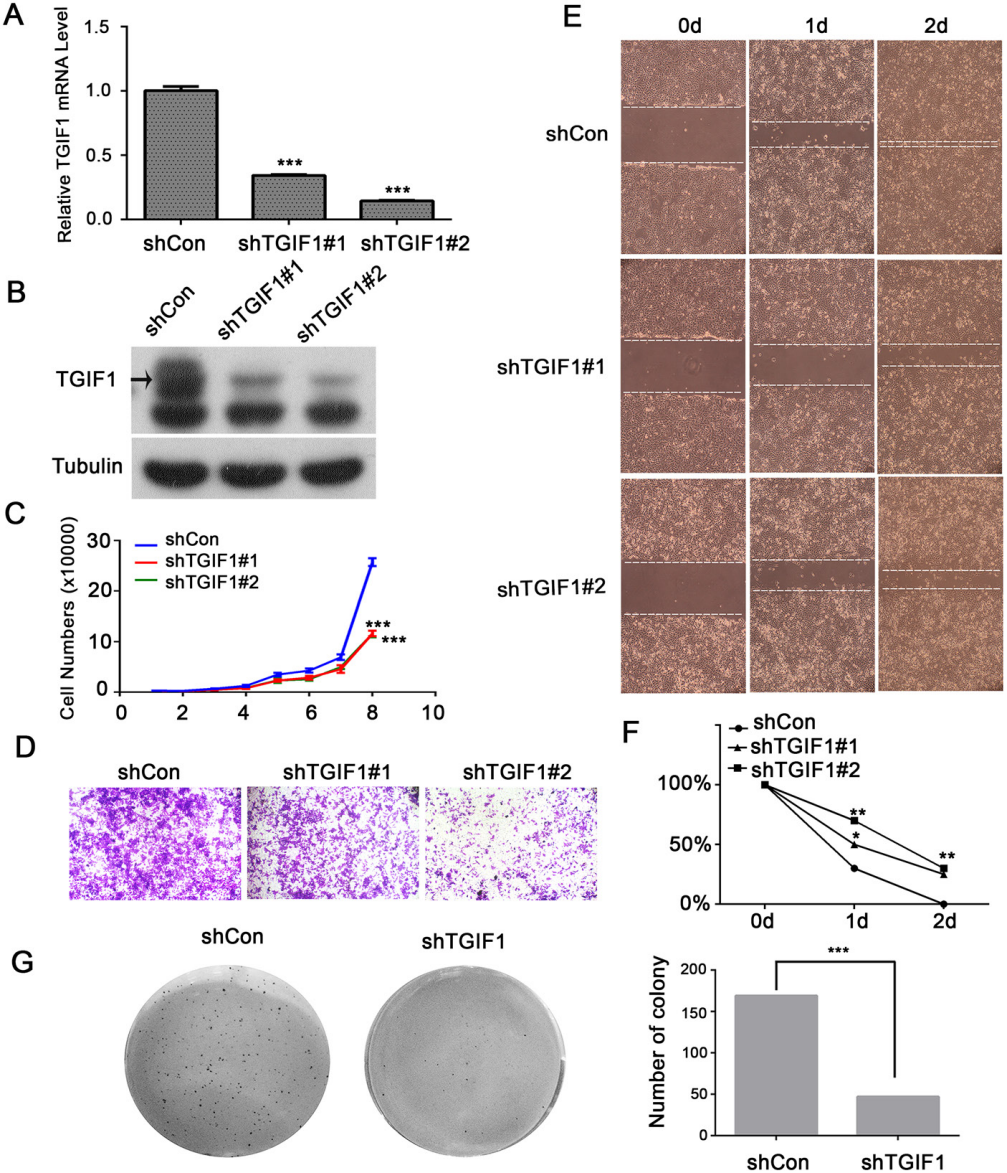
TGIF1 enhances proliferation, migration and clonogenicity of LoVo cells **(A-B)** qRT-PCR and immunoblotting showing TGIF1 knockdown efficiency in LoVo cells. **(C)** Growth curve of control and TGIF1 knockdown cells. The data are presented as the mean ± S.E. ***, *p < 0.001.* **(D)** Transwell assay using control and TGIF1 knockdown cells. After seeding cells for 24 hours, the number of migrated cells was quantified after staining with gentian violet. **(E-F)** Wound healing assay in control and TGIF1 knockdown cells. Cells were photographed every 24h after scratching. Statistical results were shown by linear graph. *, p<0.05;**, p<0.01. **(G)** Soft agar colony formation assay using control and TGIF1 knockdown cells. Images were obtained after staining with Giemsa.

To investigate TGIF1 function, the CRC LoVo cells were stably infected with lentiviruses carrying either control (shControl) or *TGIF1* shRNA (sh*TGIF1*#1 or sh*TGIF1*#2). Both qRT-PCR and immunoblotting showed that the two shRNAs efficiently decreased TGIF1 expression in the cancer cells (Figure 2A and 2B). As shown in Figure 2C, knockdown of TGIF1 in LoVo cells dramatically decreased its growth rate when compared to the control cells. Furthermore, we found TGIF1-knockdown cells showed decreased migration ability in the transwell assay (Figure 2D), which was confirmed by wound healing assay (Figure 2E). Moreover, TGIF1-knockdown cells formed significantly less colonies, as revealed by the decreased anchorage-independent growth (Figure 2F). In addition, we obtained consistent results by overexpressed TGIF1 in LoVo cells and CaCo2 cells which showed increased proliferation and migration activity (Supplementary Figures 1 and 2). We found Wnt5a and TGIF1 promotes migration, but Wnt5a has no influence on TGIF1-induced migration (Supplementary Figure 2C). These data suggest that TGIF1 enhances the proliferation, migration and anchorage-independent growth ability of colon cancer cells.

### TGIF1 promotes tumor formation *in vivo*

**Figure 3:**
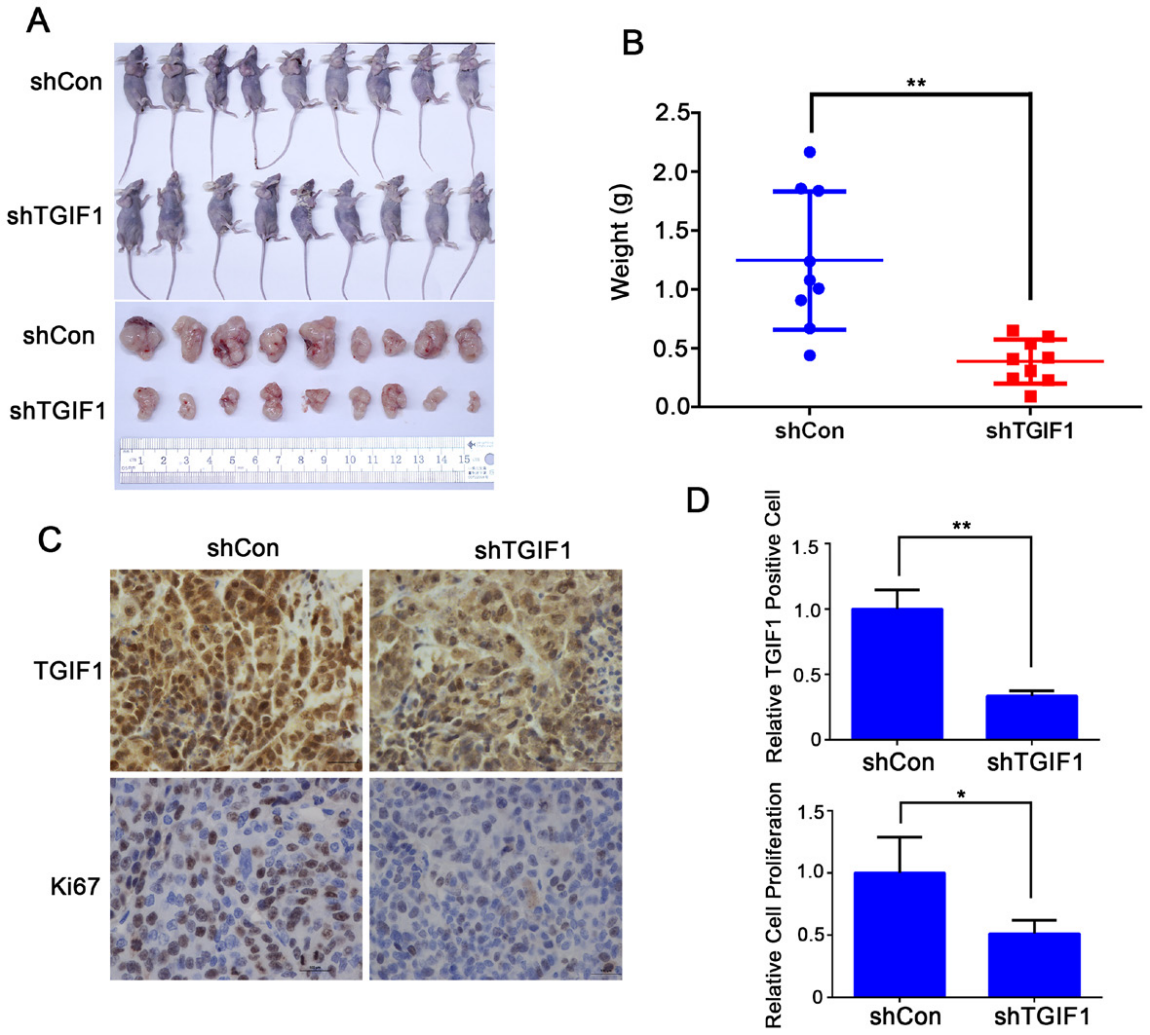
TGIF1 promotes tumor formation **(A)** Effect of TGIF1 knockdown on tumor formation in nude mice. Xenograft was collected 3 weeks after injection LoVo cells into the armpit and photographed. **(B)** The tumor weight was assessed at the terminal time. Error bars indicate S.E. of the values of each group. **, p<0.01. **(C)** Expression of TGIF1 and Ki-67 (cell proliferation marker) in xenograft tumors by immunohistochemistry. Scale bars=100 μm. **(D)** The quantification of TGIF1 positive and Ki67 positive cells in the tumors. The data are presented as the mean ± S.E. *, p<0.05;**, p<0.01.

To further investigate the role of TGIF1 in tumor formation, we evaluated tumor-promoting functions of TGIF1 in the nude mice model. LoVo cells stably transfected with lentiviruses carrying either control (shControl) or *TGIF1* shRNA were injected into the armpit of nude mice. Three weeks later, the mice were sacrificed and tumors were collected from tested mice for further analysis. We found that the average tumor size in the shTGIF1 group was much smaller than that in the control group and there was statistical difference between these two groups (Figure 3A and 3B). Immunohistochemistry analysis showed that knockdown of TGIF1 dramatically decreased cancer cell proliferation, as revealed by Ki67 staining (Figure 3C and 3D). Taken together, our findings indicate that TGIF1 promotes tumor formation *in vivo*.

### TGIF1 activates Wnt signaling in colon cancer cells

**Figure 4:**
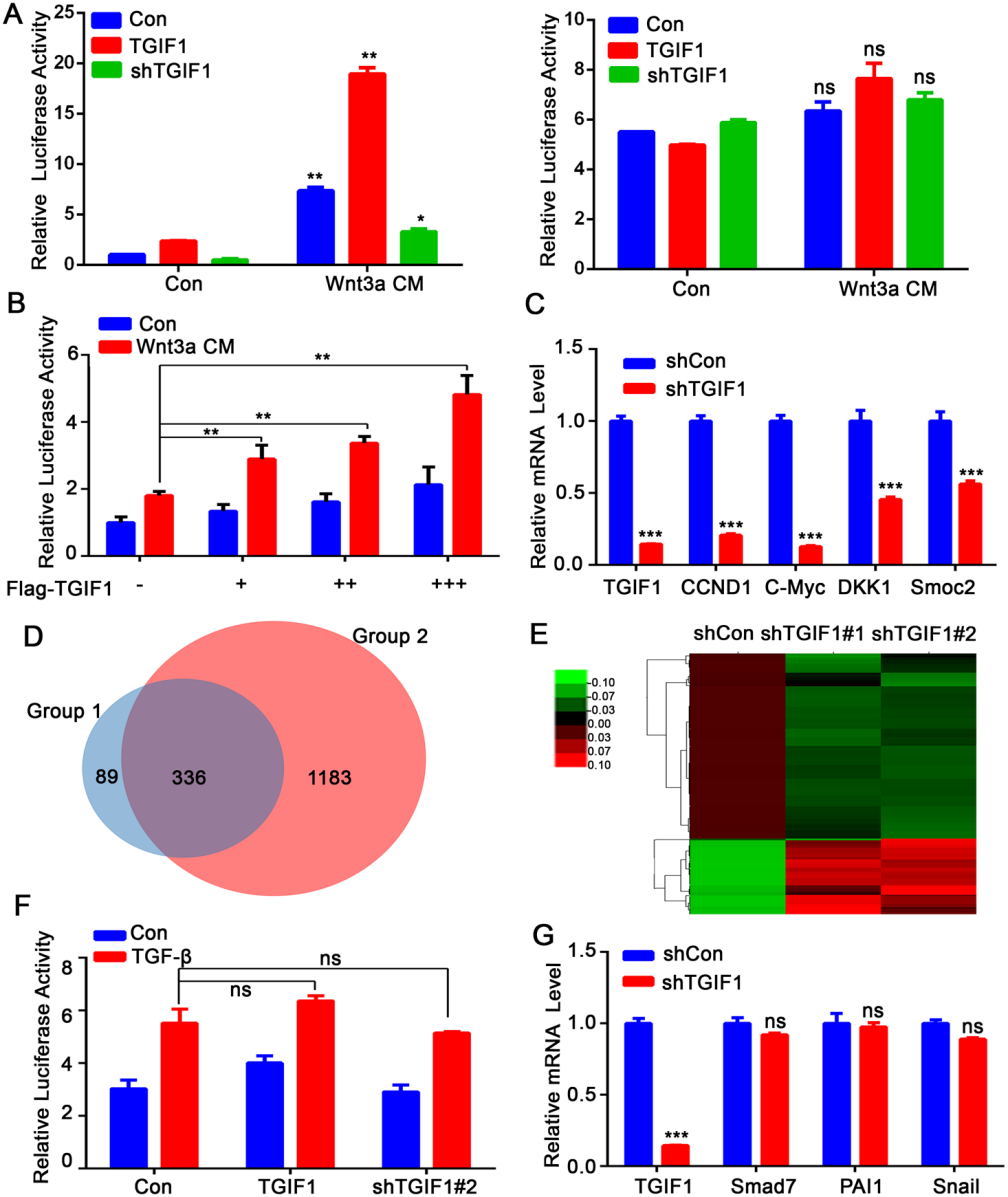
TGIF1 activates Wnt signaling in colon cancer cells **(A)** After HEK293T cells were transfected with Topflash-luciferase together with TGIF1, shTGIF1 or control vector for 24 hours, the cells were treated with Wnt3a conditional medium for another 24 hours and then harvested to measure luciferase activity. Right: Fopflash served as a negative control. **, *p <0.01*; ns, *p >0.05*. **(B)** HCT116 cells were transfected with TopFlash-luciferase and different amount of TGIF1 and harvested to measure luciferase activity after Wnt3a treatment. **, *p <0.01*. **(C)** mRNA levels of Wnt target genes (*CCND1, c-Myc, AXIN2, DKK1* and *Smoc2*) were examined by qRT-PCR in control and TGIF1 knockdown LoVo cells. *, *p <0.05;* ***, *p < 0.001.* **(D)** Venn diagram was generated to visualize overlapping genes between group 1 and group 2. Group 1 refers to shTGIF (altered in both shTGIF1#1 and shTGIF1#2) and Group 2 refers to shβ-catenin samples. **(E)** The heat map of genes expression ratio between shTGIF1#1 and shTGIF1#2. Green indicates that the gene is downregulated and red represents upregulated genes. **(F)** HCT116 cells were transfected with CAGA-luciferase together with TGIF1 expression or shRNA vector and harvested to measure luciferase activity after TGF-β (100pM) treatment. ns, *p >0.05*. **(G)** qRT-PCR analysis of TGF-β target genes (*Smad7, PAI1* and *Snail1*) in control and TGIF1 knockdown LoVo cells. ***, *p < 0.001*; ns, *p >0.05*.

In order to investigate the molecular mechanism underlying the tumor-promoting function of TGIF1, we used reporter assays to screen the potential roles of TGIF1 in regulating various signaling pathways and identified *TGIF1* as an important promoting factor of Wnt/β-catenin signaling pathway. TGIF1 enhanced the Topflash reporter activity in HEK293T cells, but had no effects on the Fopflash reporter which served as a negative control (Figure 4A). Importantly, TGIF1 could also induce Wnt-induced the Topflash reporter expression in HCT116 colon cancer cells in a dose-dependent manner (Figure 4B), in spite that Wnt signaling is constitutively active in these cells due to mutation of *β-catenin* [8]. Consistently, the mRNA levels of Wnt target genes (*CCND1, C-Myc, DKK1* and *Smock2*) were downregulated when *TGIF1* was knocked down in LoVo cells (Figure 4C). In addition, RNA-seq was used to reveal transcriptome of TGIF1. Venn diagram was generated to visualize the overlapping genes between shTGIF (altered in both shTGIF1#1 and shTGIF1#2) and shβ-catenin samples, indicating that most of the TGIF1-regulated genes are the targets of β-catenin (Figure 4D). The genes downregulated in TGIF1 knockdown cells were the Wnt targets, including *Wnt5a, NFAT5, FOSL1, RBX1* (Figure 4E). As β-catenin degradation is impaired due to loss-of-function mutations of the *APC* gene in LoVo cells [8], we speculated that TGIF1 might activate Wnt signaling downstream of the destruction complex. In contrast, alteration of TGIF1 expression did not affect the TGF-β-specific reporter CAGA-luciferase and its target gene expression in HCT116 cells which harbor TβRII mutation [32] (Figure 4F and 4G). Moreover, TGF-β has no function in cell migration (Supplementary Figure 3) because most colon cancer cells are resistant to TGF-β signaling due to mutation in its receptors or Smad proteins [32, 33]. We therefore conclude that the function of TGIF1 in CRC progression is via promoting Wnt signaling, but independent of TGF-β signaling.

### TGIF1 homeodomain is essential for promoting Wnt signaling

**Figure 5:**
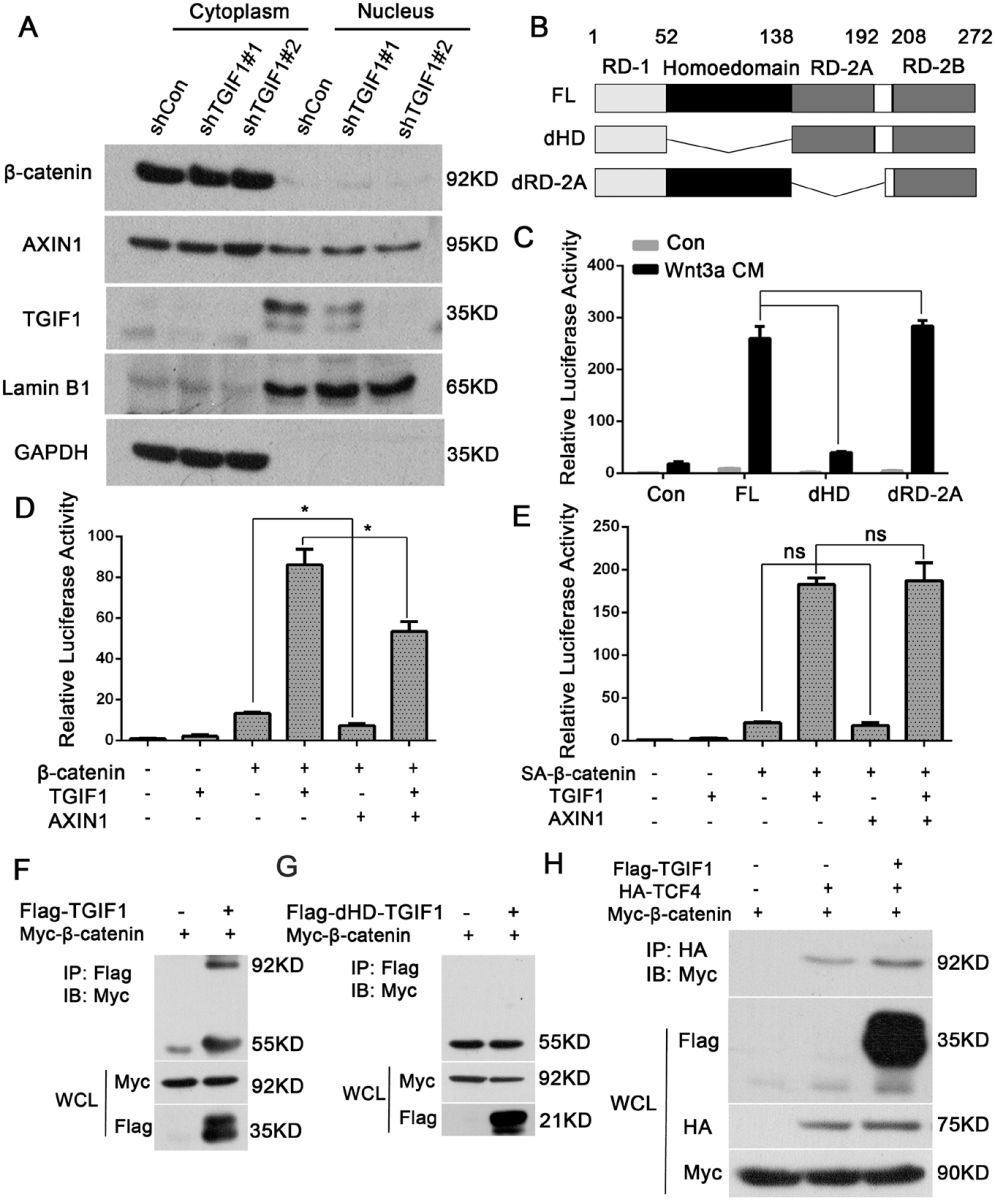
The TGIF1 homeodomain is essential for promoting Wnt signaling **(A)** Nuclear and cytoplasmic fractions were separated from LoVo cells and subjected to immunoblotting with the indicated antibodies. **(B)** Diagrams showing TGIF1 domain deletion constructs. FL for full length; dHD for homeodomain deletion and dRD-2A for RD-2A domain deletion. **(C)** Wild-type TGIF1 and various deletion constructs were co-transfected with Topflash-luciferase into HEK293T cells. After 24 hours, the cells were treated with Wnt3a conditional medium for another 24 hours and then harvested to measure luciferase activity. ***, *p < 0.001*; ns, *p>0.05*. **(D-E)** HEK293T cells were transfected with Topflash-luciferase together with TGIF1, AXIN1, wild-type β-catenin or SA-β-catenin as indicated. At 48 hour post-transfection, the cells were harvested for luciferase assay. *, p <0.05; ns, p>0.05. **(F-G)** HEK293FT cells were co-transfected with constructs carrying β-catenin, TGIF1 and dHD-TGIF1 as indicated for 48h before they were harvested. Cell lyses were precipitated with anti-Flag antibody, followed by anti-Myc immunoblotting (IB) to detect the interaction between β-catenin and TGIF1. IP, immunoprecipitation; WCL, whole cell lysate. **(H)** HEK293FT cells were co-transfected with constructs carrying β-catenin, TGIF1 and TCF4 as indicated for 48h before they were harvested. Cell lyses were precipitated with anti-HA antibody, followed by anti-Myc immunoblotting (IB) to detect the interaction between β-catenin and TCF4.

A previous study has suggested that TGIF1 could sequestrate Axin1/2 in the nucleus to prevent the assembly of the destruction complex, thereby promoting Wnt signaling in breast cancer cells [30]. However, we found that knockdown of TGIF1 had no effects on the distribution of both β-catenin and Axin2 in the nucleus (Figure 5A), suggesting the existence of a different mechanism underlying Wnt activation by TGIF1 in colon cancer cells. Immunofluorescence shows consistent results (Supplementary Figure 4A and 4B). TGIF1 contains two separate recruitment domains, one of which binds directly to DNA and the other interacts with TGF-β-activated Smad proteins and HDAC1 to repress transcription [21, 34]. To investigate the function of these domains, we generated different TGIF1 deletion constructs (Figure 5B), and found that deletion of the DNA-interacting homeodomain significantly disrupted its ability to activate Wnt signaling (Figure 5C). In addition, the homeodomain was required for the promoting effect of TGIF1 on migration (Supplementary Figure 1). Although overexpression of Axin1 could decrease β-catenin-induced Topflash reporter activity, TGIF1 was still able to enhance Wnt signaling with Axin1 overexpression (Figure 5D), suggesting that TGIF1 may function independently of Axin1. Moreover, we found TGIF1 could also promote the active β-catenin (SA-β-catenin) induced Topflash-luciferase reporter (Figure 5E). As SA mutation renders β-catenin resistant to degradation by the destruction complex [35], we therefore reason that TGIF1 may promote Wnt signaling downstream the destruction complex by directly binding to DNA. Unfortunately, we were unable to test this as we did not found any quality TGIF1 antibody for chromatin immunoprecipitation. Next, we tested whether TGIF1 interacts with β-catenin or promotes the interaction between β-catenin and TCF4. The immunoprecipitation experiment shows that TGIF1 interacts with β-catenin (Figure 5F), while deletion of the homeodomain lost this interaction (Figure 5G). We found TGIF1 increased interaction of β-catenin and TCF4 (Figure 5H). In addition, after knock down TGIF1, interaction of β-catenin and TCF4 was decreased (Supplementary Figure 4C). Therefore, TGIF1 could promote Wnt signaling either through direct DNA binding or through its interaction with β-catenin that enhances the formation of the β-catenin/TCF4 complex.

### TGIF1 forms a positive feedback loop to sustain high Wnt activity

**Figure 6:**
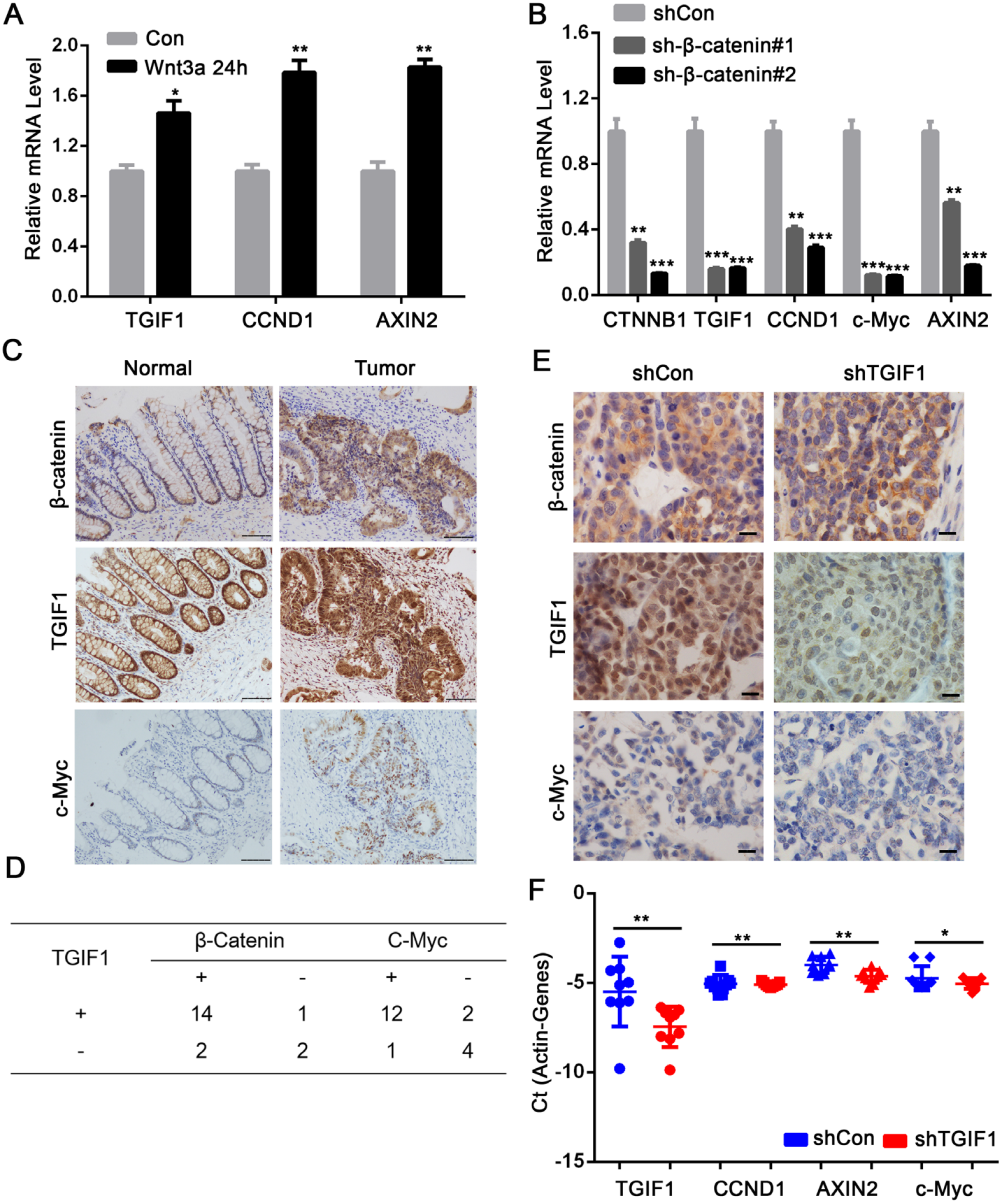
TGIF1 forms a positive feedback loop to sustain Wnt signaling **(A)** HCT116 cells were treated with Wnt3a for various times and then harvested for qRT-PCR to assess mRNA expression of TGIF1, CCND1 and AXIN2. **(B)** After LoVo cells were transfected with shControl and *β-catenin* shRNA for 24 hours, mRNA expression of β-catenin, TGIF1 CCND1, c-Myc and AXIN2 were examined by qRT-PCR. *, *p <0.*05; **, p <0.01; ***, p<0.001. **(C)** Protein expression of β-catenin, TGIF1 and c-Myc in CRC tissues (Tumor) and paired normal tissues (Normal) were tested by Immunohistochemistry. Scale bars=100μm. **(D)** Expression correlation between TGIF1 and β-catenin or TGIF1 and c-Myc in CRC samples. **(E)** Immunohistochemistry was performed in the xenografts derived from control and TGIF1 knockdown LoVo cells. Scale bars=100 μm. **(F)** Expression of Wnt target genes (*CCND1*, *AXIN2* and *c-Myc*) was performed in the xenografts derived from control and TGIF1 knockdown LoVo cells. Error bars indicate S.E. of the values of each group. *, *p<0.05*; **, *p<0.01*.

Previous work identified TGIF1 as a Wnt target gene [30], which was confirmed in our study using qRT-PCR in HCT116 cells (Figure 6A). Consistently, depletion of *β-catenin* led to downregulation of TGIF1 and other Wnt target genes including *CCND1*, *c-Myc* and *AXIN2* in LoVo cells (Figure 6B). Moreover, overexpressed dominant TCF4, TGIF1 and Wnt target genes mRNA level decreased (Supplementary Figure 4D). Furthermore, in the CRC tissue, β-catenin and TGIF1 were significantly upregulated in the nuclei of cancer cells (Figure 6C). The Wnt target gene *c-Myc* was also highly expressed in tumor tissues compared to controls, indicating the hyperactivation of Wnt signaling in cancer tissues (Figure 6C). The abnormal expression of TGIF1 was significantly correlated to c-Myc (χ^2^=0.32, r=0.62, *p=0.017*) and β-catenin (χ^2^=0.34, r=0.48, *p=0.018*) (Figure 6D). In contrast, in the xenografts derived from TGIF1 knockdown cells, β-catenin was still found in the nucleus but the high expression of c-Myc was not observed (Figure 6E). Moreover, mRNA levels of the Wnt target genes *CCND1*, *AXIN2* and *c-Myc* were dramatically decreased in the tumor with low TGIF1 levels (Figure 6F). Together, these results strongly indicate that TGIF1 plays a critical role in Wnt signaling by forming a positive feedback loop to sustain high Wnt activity in colon cancer progression. Schematic model was in Supplementary Figure 5.

## DISCUSSION

It is previously reported that TGIF1 plays important roles in embryonic development, stem cell self-renewal/quiescence, adipocyte differentiation and vascularization [27–29, 36]. Recent studies further indicate the pivotal function of TGIF1 in tumorigenesis. TGIF1 was shown to enhance the progression of oral squamous cell carcinoma by repressing TGF-β signaling [37], while in breast cancer and lung cancer, TGIF1 can promote tumor growth through activating Wnt/β-catenin signaling independently of TGF-β signaling [30, 31]. The functions of TGIF1 in leukemia, gastric cancer and ovarian cancer were also reported [38–40]. However, it remains elusive whether TGIF1 has a general tumor promoting function in colorectal cancer. Here we identified TGIF1 as a novel tumor promoter in colon cancer, and demonstrated that TGIF1 promotes proliferation and migration of colon cancer cells. Importantly, we showed that TGIF1 functions through activating Wnt/β-catenin signaling in colon cancer cells.

Our study revealed that TGIF1 was highly expressed in colorectal cancer and promoted proliferation and migration of cancer cells. Moreover, TGIF1 activated Wnt signaling and was critical for the expression of the Wnt target genes in colorectal cancer cells, indicating that the tumor promoting function of TGIF1 is mediated by Wnt signaling. It has been well documented that TGIF1 represses TGF-β signaling [22]. However, accumulating evidence indicates that TGIF1 has TGF-β-independent functions.

TGIF1 can activate Wnt signaling in breast and lung cancer cells, but the mechanism is not very clear. Zhang et al showed that TGIF1 broke the destruction complex and then drove AXIN1/2 to the nucleus, thus affecting β-catenin abundance in breast cancer cells [30]. However, we found that knockdown of TGIF1 has no obvious effects on β-catenin and Axin2 protein levels in the nuclei of colorectal cancer cells. Instead, the homeodomain of TGIF1 is essential in promoting Wnt signaling in colorectal cancer cells, suggesting that TGIF1 may activate Wnt signaling through a different mechanism in different cell types. Indeed, our data showed that the TGIF1 could interact with β-catenin via its homeodomain and increase the interaction between β-catenin and TCF4. As TGIF1 can interact with histone deacetylase [21, 22], it is also possible that TGIF1 directly regulates Wnt target expression by regulating chromatin accessibility. These detailed mechanism underlying the role of TGIF1 in Wnt signaling merits future investigations.

Our results suggest that TGIF1 may form a positive feedback loop to amplify Wnt signaling. In CRC tissues, the nuclear β-catenin is greatly elevated, which is accompanied by enhanced TGIF1 expression, which in turn can maintain high Wnt signaling activity and thus promote colorectal cancer development. Otherwise, β-catenin overexpression may not be insufficient to support high expression of Wnt target genes in the absence of TGIF1. Our findings unravel a positive feedback loop in colorectal cancer development. Therefore, disruption of this loop by inhibition TGIF1 could be a therapeutic strategy to treat colorectal cancer.

## MATERIALS AND METHODS

### Tumor samples and cell lines

A total of 19 pairs of CRCs and the corresponding nontumorous tissues were obtained from Peking University Third Hospital in 2014 which were approved by the Ethics Committee. Fresh samples were derived from patients undergoing surgical resection, and then frozen immediately into liquid nitrogen. Colorectal cancer cell lines (LoVo and HCT116) were purchased from China Infrastructure of Cell Line Resources in 2015. Cells were maintained in McCoy’s 5a medium (HCT116), or DMEM (LoVo, HEK293FT, and HEK293T) supplemented with 10 % FBS at 37°C incubator containing 5 % CO_2_.

### RNA extraction and real-time PCR analysis

Total RNA was extracted from CRC tissues and cell lines using Trizol reagent (Invitrogen). cDNA was synthesized using 1ug RNA by ReverTra Ace -α- kit (TOYOBO). Amplification reactions were performed in a 15 μL volume of Realtime PCR Master Mix (TOYOBO) on LC480 (Roche Applied Science) system. All the primers used are listed in Table 1.

**Table 1:** Primers for RT-PCR

Gene	Forward primer	Reverse primer
*ACTIN*	GTACCACTGGCATCGTGATGGACT	CCGCTCATTGCCAATGGTGAT
*TGIF1*	GACATTCCCTTGGACCTTTCT	TACAGCCAATCCCGAAGAATC
*CCND1*	CTGGCCATGAACTACCTGGA	CTCCGCCTCTGGCATTTTGG
*C-Myc*	TCTCCTTGCAGCTGCTTAG	GTCGTAGTCGAGGTCATAG
*AXIN2*	AGTGTGAGGTCCACGGAAAC	CTTCACACTGCGATGCATTT
*DKK1*	TCCCCTGTGATTGCAGTAAA	TCCAAGAGATCCTTGCGTTC
*SMOC2*	ACAATGAATCACGCAACCACTA	CAAAGTATCACACCAACGACAGT
*SMAD7*	ACCCGATGGATTTTCTCAAAC	GCCAGATAATTCGTTCCCCC
*PAI1*	GAGACAGGCAGCTCGGATTC	GGCCTCCCAAAGTGCATTAC
*SNAIL1*	TCGGAAGCCTAACTACAGCGA	AGATGAGCATTGGCAGCGAG

### Constructs

TGIF1 coding sequence were PCR-amplified using HEK293T cDNA as a template, and then cloned into pCDNA3.1 vector containing a Flag tag between KpnI/XhoI sites. All deletion constructs (1-52:138-272(dHD), 1-138:200-272(dRD-2A)) were obtained by the standard PCR overlap extension technique. The plasmid encoding TGIF1 was denatured and annealed to a mutagenic primer. The resulting fragments were connected in a final overlap PCR. The chimerical overlap PCR fragments were inserted into the vector between KpnI/XhoI sites. The sequence of all mutants was verified by sequencing.

### Immunoblotting, immunoprecipitation, immunohistochemistry, immunofluorescence and antibodies

For immunoblotting , cells were lysed in TNE buffer (20 mM Tris-HCl, pH 7.4, 150 mM NaCl, 2 mM EDTA, 1 % Triton X-100) with freshly added proteinase inhibitor cocktail (Roche Applied Science) for 30 min at 4°C. After centrifugation, the supernatants were resolved by SDS-PAGE. For immunoprecipitation, one-tenth of the cell lysate was preserved as whole cell lysate, and the remaining cell lysate was incubated with 2 μg of antibodies and 30 μl of protein A-Sepharose beads (Zymed Laboratories Inc.) overnight at 4°C. The beads were washed three times with TNE buffer and then analyzed using immunoblotting. For immunohistochemistry, colorectal cancer tissues and paired nontumorous tissues were fixed in formalin, embedded in paraffin, and cut into 5-μm-thick consecutive sections. After deparaffin and antigen recovery (in sodium citrate solution, pH 6.0, 15 min, 98°C), samples were denatured for 30min at room temperature in methanol contained 0.003 % H_2_O_2_, blocked for 30 min at 37°C in PBS contained 0.3 % Triton X-100 and 5 % BSA, followed by incubating with primary antibody at 4°C overnight, and finally the secondary antibody for 30 min at 37°C. Images were obtained with Nikon. For immunofluorescence, cells were sequentially treated with 4% paraformaldehyde for 10 min, 0.5% Triton X-100 for 5 min, 0.5% BSA for 30min, the primary antibody overnight at 4°C, and finally the secondary antibody for 1 h in the dark. The nuclei were counterstained with DAPI (Sigma). Images were obtained with confocal Olympus FluoView 1200 microscope. The following antibodies were used: TGIF1 (sc9084), β-catenin (ab32572), C-Myc (ab32072), tubulin (Proteintech 66031-1-Ig), LaminB1 (sc-56143), AXIN1/2 (sc-14029) and TCF4(sc-8631).

### TGIF1 knockdown

Specific shRNAs for *TGIF1* were obtained from Sigma (shRNA lentiviral transduction particles TRCN0000020149 and TRCN0000020151). Lentivirus was produced by transfection of HEK293FT cells with lentiviral vectors using Lipofectamine 2000 (Invitrogen) according to manufacturer’s instructions. After 72h, the supernatant was collected and filtered through a 0.45 μm filter (Millipore). The virus-containing supernatants were added into plates of LoVo cells in the presence of 8 μg/ml polybrene. After incubation for 6 h at 37°C, equal volume of culture medium was added and further incubated overnight before the supernatants were discarded. After 48h, cells were cultured with medium containing puromycin (1 μg/ml) to select cells with stable virus integration. Cells were collected to test knockdown efficiency by immunoblotting.

### Cell proliferation assay

Cells were seeded into a 24-well plate at a density of 3000 cells/well and cultured with medium containing puromycin (1 μg/ml). Cell numbers were counted at the indicated time points.

### Transwell migration assay

Cell migration was assessed using 24-well inserts with 8 μm pores (Corning). Ten thousand cells suspended in 300 μL culture medium with 1 % FBS were placed inside each insert, while 500 μL culture medium with 10 % FBS were added to lower chamber as a chemoattractant. After 24 hours, migrated cells were stained with gentian violet, photographed and quantified.

### Wound healing assay

Cells were seeded in a 6-well plate at a density of 1 x 10^6^ cells/well, and then cultured to proper density. In order to verify reliability, cells were seeded at the same density to create a confluent monolayer onto the well as fast as quickly. The scratch in different groups was examined under microscope. Before scratching, cells were treated with 10μg/ml mitomycin C for 2h in order to suppress proliferation. After scratching, cells were photographed every 24 hours.

### Soft agar colony-formation assay

Agarose (1.2 %) (Promega, V2111-5g) and 20 % FBS were mixed at a ratio of 1:1. Then 1.4mL mixture was added in 6-well plate for 30 min at 4°C. After solidation, 1mL 0.3 % agarose containing 3000 cells were plated in 6 well plate. Cells were cultured for 10-14 days. 100 μL culture medium was added every 3 days. Images were obtained after staining with Giemsa.

### Luciferase reporter analysis

HEK293T and HCT116 cells were seeded in 24-well plates and transfected with Vigo transfection Reagent at 60-80 % confluence. pRenilla-TK vector was used as an internal control. At 48h post-transfection, cells were collected and the reporter activity was measured using Luciferase assay system (Promega).

### *In vivo* tumor formation assay

Two million cells in 0.1 mL PBS were subcutaneously injected into the left axilla of female nude mice (4 weeks old, nine mice per group). The weight of nude mice was measured every 7 days for 3 weeks. The tumor weight was assessed at the terminal time. The protocols for *in vivo* animal experiment were approved by the Ethics Committee of Peking University Health Science Center.

### Nuclear and cytoplasmic proteins extraction

One million cells were harvested with trypsin and then centrifuged at 500 g for 5 minutes. Nuclear and cytoplasmic lysates were prepared using the NE-PER Nuclear and Cytoplasmic Extraction Reagent Kit (Thermo Scientific, Waltham, MA) according to manufacturer’s instructions.

### Statistical analysis

All the experiments were independently repeated at least three times. Statistical analysis was performed with SPSS version 20. The values were presented as mean ± S.D., and the significance between means was calculated using Student’s t test. χ2 test was used to assess the correlation between TGIF1 and c-Myc or β-catenin. *p* value less than 0.05 was considered as statistically significant (* for *p<0.05*, ** for *p<0.01* and *** for *p<0.001*).

## SUPPLEMENTARY MATERIALS FIGURES



## Abbreviations

TGIF1: TGFB induced factor homeobox 1
CRC: colorectal cancer
TGF-β: transforming growth factor-β
GSK-3β: glycogen synthase kinase 3β
TCF/LEF: T cell factor/lymphoid enhancer binding factor
HDACs: histone deacetylases
RA: retinoic acid
TCGA: The Cancer Genome Atlas
CCND1: Cyclin D1
CTNNB1: catenin beta 1
SMOC2: SPARC related modular calcium binding 2
DKK1: dickkopf WNT signaling pathway inhibitor 1
SNAIL1: snail family transcriptional repressor 1
PAI1: serpin family E member 1
CM: conditional medium
shRNA: short hairpin RNA
qRT-PCR: quantitative real-time polymerase chain reaction
