# Supplementary material for: TGFβ induced factor homeobox 1 promotes colorectal cancer development through activating Wnt/β-catenin signaling
Source: Oncotarget. 2017 Jul 26;8(41):70214–25. doi: 10.18632/oncotarget.19603 (PMC5642548; doi:10.18632/oncotarget.19603)
Supplement: Supplementary file 1 [file oncotarget-08-70214-s001.pdf]

TGFβ induced factor homeobox 1 promotes colorectal cancer development through activating Wnt/β-catenin signaling

SUPPLEMENTARY MATERIALS

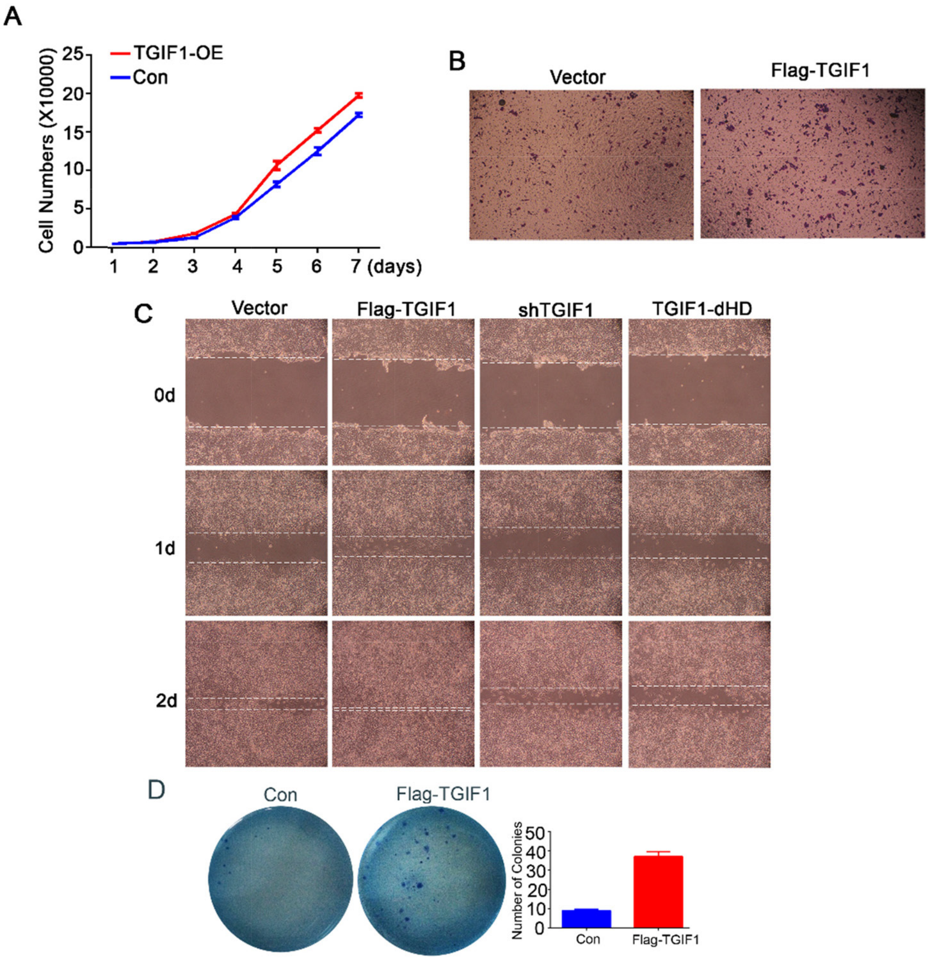

Supplementary Figure 1: (A) Growth curve of control and TGIF1 overexpressed LoVo cells. **(B)** Transwell assay using control and TGIF1 knockdown cells. After seeding cells for 24 hours, the number of migrated cells was quantified after staining with gentian violet. **(C)** Wound healing assay in control and TGIF1 overexpression, TGIF1 knockdown, and deletion of TGIF1 homeodomain cells. Cells were photographed every 24h after scratching. **(D)** Soft agar colony formation assay using control and TGIF1 overexpressed cells. Images were obtained after staining with Giemsa.

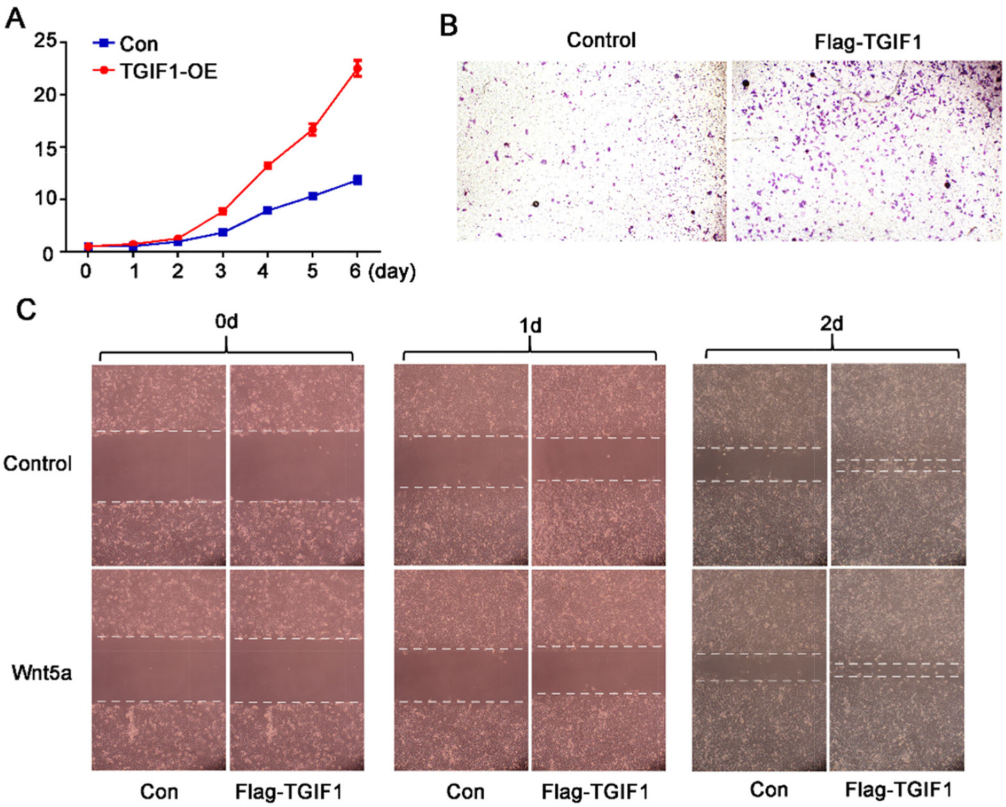

Supplementary Figure 2: (A) Growth curve of control and TGIF1 overexpressed CaCo-2 cells. **(B)** Transwell assay using control and TGIF1 overexpressed CaCo-2 cancer cells. After seeding cells for 24 hours, the number of migrated cells was quantified after staining with gentian violet. **(C)** Wound healing assay in control and TGIF1 overexpressed CaCo-2 cancer cells with Wnt5a treatment. Cells were photographed every 24h after scratching.

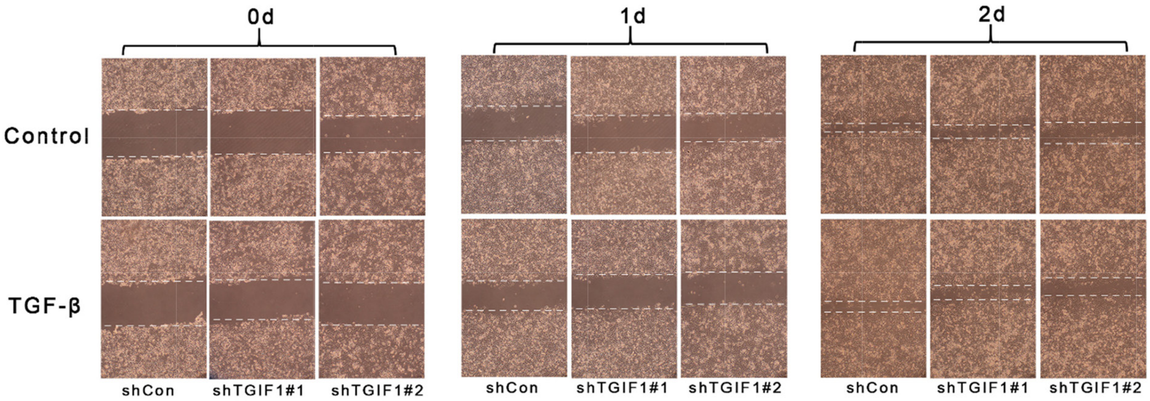

Supplementary Figure 3: Wound healing assay in control and TGIF1 knockdown LoVo cells under TGF-β (100pM). Cells were photographed every 24h after scratching.

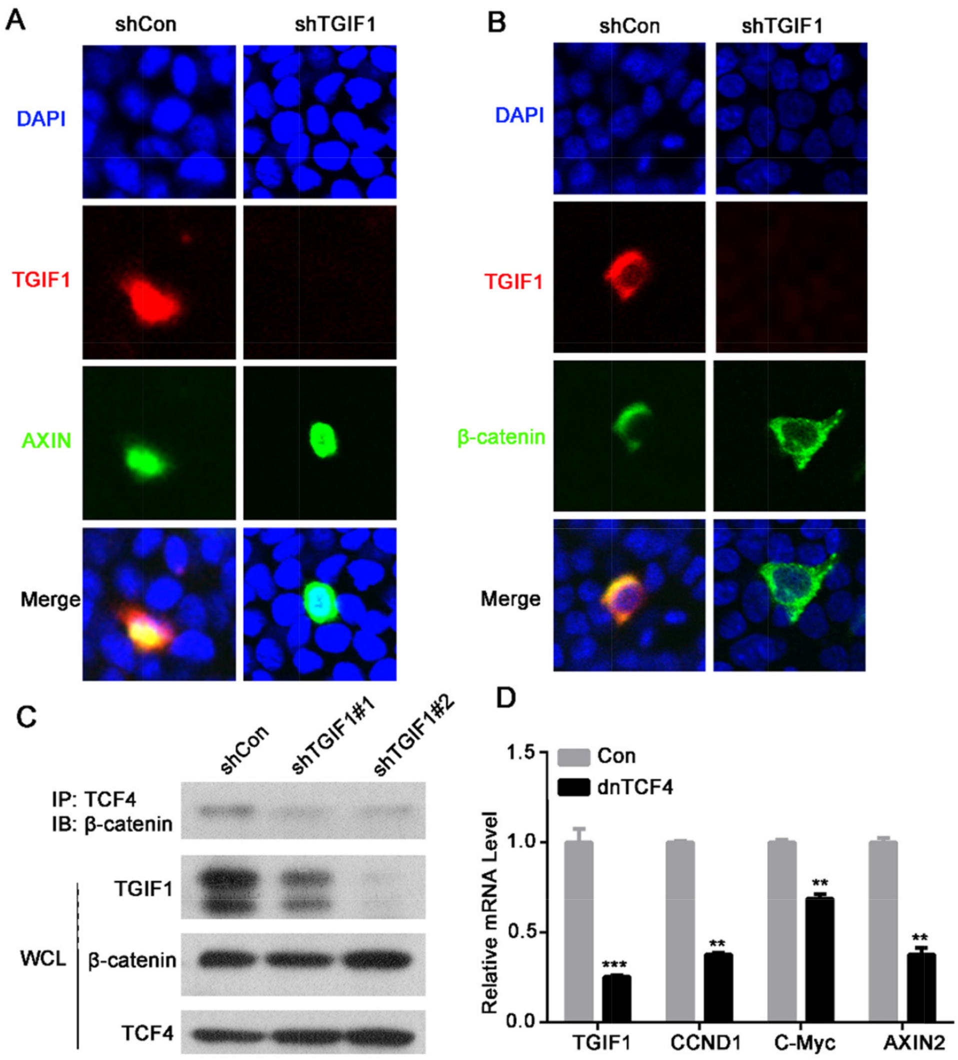

Supplementary Figure 4: (A-B) LoVo cells were harvested for anti-TGIF1/AXIN/β-Catenin immunostaining. **(C)** LoVo cell lyses were precipitated with anti-TCF4 antibody, followed by anti-β-catenin immunoblotting (IB) to detect the interaction between β-catenin and TCF4. **(D)** mRNA levels of Wnt target genes (*CCND1, c-Myc, AXIN2*) were examined by qRT-PCR in control and dominant TCF4 HCT116 cells.

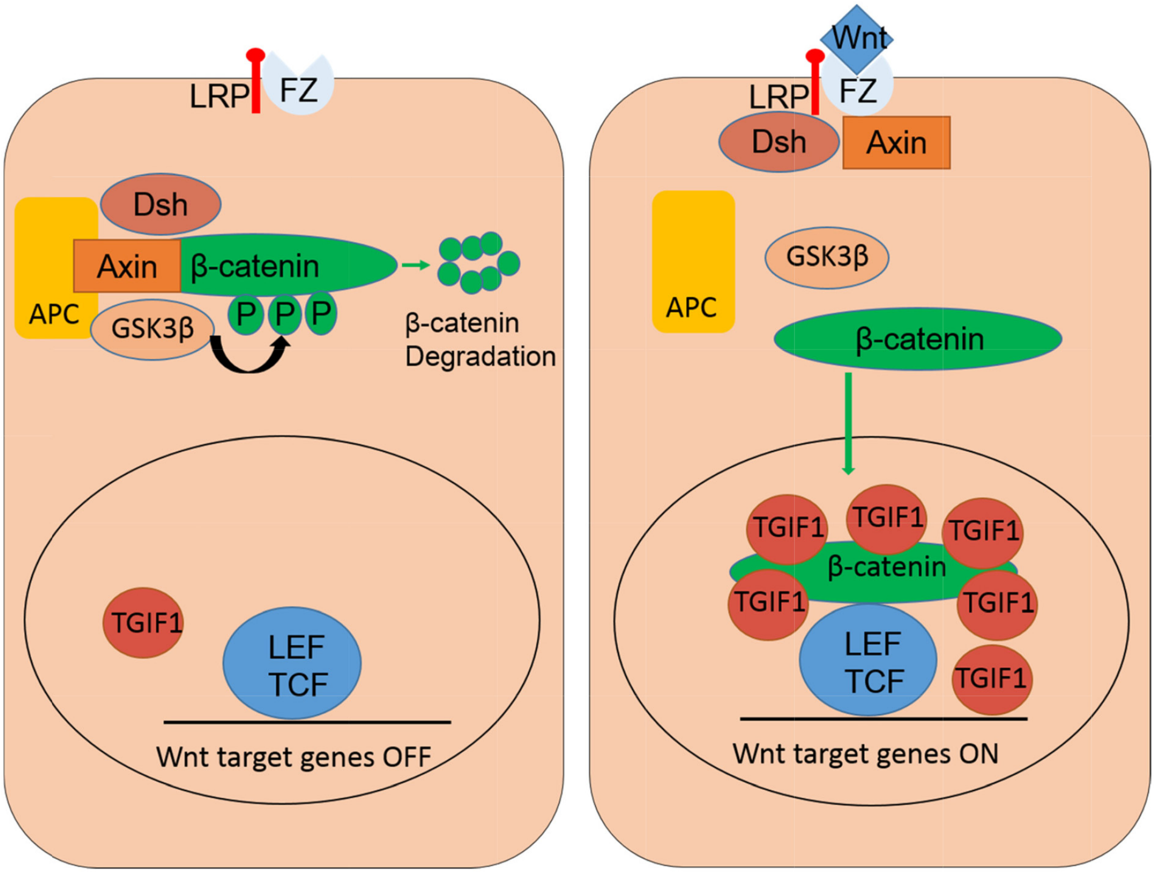

Supplementary Figure 5: (Left) In the absence of Wnt stimulation, β-catenin levels are kept at a minimum through the destruction complex. (Right) In the presence of Wnt stimulation, the destruction complex is destabilized and TGIF1 upregulated, and β-catenin accumulates in the nucleus to activate transcription of Tcf target genes. In addition, TGIF1 could activate Wnt signaling through direct DNA binding, but more evidence waits to be provided.
